# Frailty and innovative participatory rehabilitation

**DOI:** 10.1016/j.jnha.2023.100012

**Published:** 2024-01-01

**Authors:** Martin Skoumal, Martina Honegger, Regina Roller-Wirnsberger

**Affiliations:** a Austrian Pension Insurance, Department for Scientific Research in Rehabilitation, Friedrich-Hillegeist-Straße 1, 1021 Vienna, Austria; b Medical University of Graz, Department of Internal Medicine, Research Unit Old Age Medicine and Lifelong Health, Auenbruggerplatz 15, 8036 Graz, Austria

**Keywords:** Rehabilitation, Frailty, Individual resilience, Social participation

## Abstract

This Mini-Review showcases the latest evidence on rehabilitation opportunities for older people with multimorbidity and frailty. There is growing evidence, that a person-centered and contextualized rehabilitation approach may offer benefits, not only in the context of preserving mobility, but especially targeting social participation. Modern rehabilitation aligns with the bio-psycho-social model of the International Classification of Functioning, Disability and Health (ICF), emphasizing the individual and collaboratively determined definition of personalized rehabilitation goals at the activity and participation level.

Further studies are warranted to evaluate objective outcome-measurement tools within the domains of activity and participation.

Frailty has become a major clinical and public health concern. It is characterized by the reduced capacity of multiple physiological systems leading to an increased vulnerability to stressors [1]. A recent editorial postulates a comprehensive biological, clinical and real-world view on management of frailty, thereby increasing resilience and allowing older people to actively participate within their environments [2]. Evidence indicates that resilience is independently linked to the onset of frailty, with particular emphasis on domains such as vitality and locomotor function [3]. Beyond these early stages of frailty, multifaceted interventions may target improved outcome of functional performance of older multimorbid people with frailty. In a recent article published in the JNHA, experts provided a summary of the current clinical evidence for managing frailty. The article underscored the need for integrated care processes aimed at fostering resilience in ageing societies [4].

The World Health Organization's (WHO) "Rehabilitation 2030" initiative has elucidated the role of rehabilitation as an integral component of integrated care, emphasizing its value and societal significance [5]. Rehabilitation should focus on minimizing activity limitation and maximizing social participation, even when body structure and function cannot be restored to premorbid levels [6]. In this Mini-Review authors try to give a brief overview on current evidence and future perspectives for rehabilitation in multimorbid citizens of advanced age with geriatric syndromes and/or frailty, summarized under the umbrella term of “geriatric rehabilitation” (GR) [7].

## 1 Rehabilitation for geriatric patients - current evidence for person-centered rehabilitation goals

The European Geriatric Medicine Society (EuGMS) has recently launched a consensus document on GR [8]. This expert-opinion-based consensus advises integration of Comprehensive Geriatric Assessment (CGA) in rehabilitation planning. CGA allows a solid bio-psycho-social evaluation for a meaningful “shared decision making” (SDM) process together with older adults at the start of rehabilitation [9]. SDM facilitates the development of a person-centered and context-sensitive rehabilitation plan, tailored towards a patient's individual goals. The process of supporting a patient’s adherence towards the rehabilitation program and the monitoring of the therapeutic progress are critical elements for fostering a sustainable and participatory rehabilitation process [10]. Van der Kluit et al. report a favourable personal perception of rehabilitation outcomes compared to more “generalized” patient reported outcome measures (PROMs) [11].

**Fig. 1:**
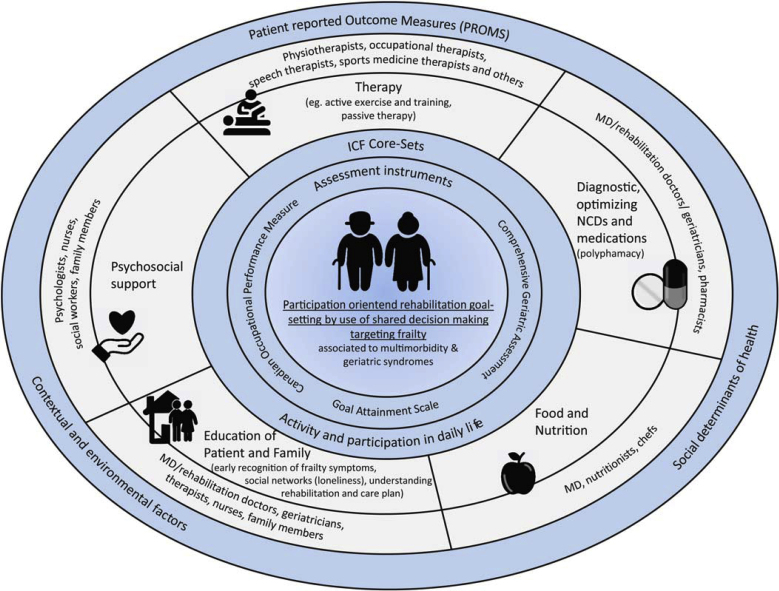
Ecosystem for innovative participatory geriatric rehabilitation

Fig. 1 describes the structural ecosystem for innovative participatory rehabilitation. In addition to the recent recommendations put forth by geriatricians, most data on rehabilitation have been gathered by experts in the field of rehabilitation. In this context, a systematic review addressing the rehabilitation needs of older multimorbid adults during GR underlines the usefulness of another instrument at admission to rehabilitation, the “Goal Attainment Scale” (GAS) [12]. Moreover, while the Canadian Occupational Performance Measure (COPM) has been discussed in the context of GR, it is important to note that there is currently a lack of validation for scores specifically targeting frailty [13]. Levak et al. also stressed the impact of using person-centered and personally meaningful goals in GR, especially when targeting people's quality of life, well-being and self-efficacy [14]. Therefore, “social participation” and “social determinants of health” can be seen as “the core-principle and target” of GR.

This concept is also mirrored by the International Classification of Functioning, Disability and Health (ICF) in rehabilitation medicine. The ICF Core-Set has been formulated based upon the bio-psycho-social model of health, enabling the systematic tracking of rehabilitation outcomes at a more structured level [15].

## 2 Multifaceted rehabilitation to address frailty

The overall aim of rehabilitation in older multimorbid adults at risk or already presenting with frailty, is to provide the right type of therapy, at the right time, in the right setting for the individual patient [16].

This includes geriatric syndromes such as falls, malnutrition, sarcopenia and many others, all aimed at enabling social participation. When using ICF‘s activity and participation domains, priorities can be identified for the rehabilitation program. During the admission to rehabilitation, patients’ focus is reported more on areas of coping with activity limitations and less on limitations in participation [17]. As recently shown in the RESORT study, frailer GR inpatients suffer from more complicated diseases and impaired nutritional, physical, and psychological markers. Additionally, cognitive impairment, delirium, comorbidity and anxiety at admission predicted worsening frailty during rehabilitation [18]. The authors emphasize that within a geriatric rehabilitation care plan, all domains affected by or influencing frailty must be promptly addressed. A systematic review and meta-analysis evaluated various protocols involving tailored nutritional interventions, both with or without exercise interventions, in the rehabilitation of geriatric patients. A key finding of the study was that oral nutritional supplements [7] during the rehabilitation of frail older adults improve nutritional outcomes, particularly evident in increased protein intake and elevated serum albumin levels post rehabilitation [19]. The improvement of functional outcomes indicates benefits for protein supplementation in this population.

As for nutrition, physical intervention needs to be tailored towards individual goals and capacities. Interestingly, factors such as higher disease burden, cognitive impairment, anxiety and depression scores, malnutrition and advanced stages of frailty and sarcopenia have been identified as independent contributors to a reduced frequency of physiotherapy (PT) and exercise training during the rehabilitation process. In in-house rehabilitation settings, older age, female sex, musculoskeletal reason for admission, greater independence in activities of daily living and handgrip strength were associated with a higher PT frequency [20]. Consensus recommendations highlight the benefits of moderate intensity physical activity five times a week for frail older subjects with additional intake of vitamin D and protein. The efficacy of this strategy has been validated in community dwelling older adults with physical frailty and sarcopenia as demonstrated by a recent multicenter trial in Europe known as the SPRINTT study. The study specifically targeted individuals with short physical performance battery scores ranging from 3 to 7. The study provides evidence that a multicomponent intervention encompassing nutrition and physical exercise components was associated with a reduction in the incidence of mobility restrictions among older adults. Community-based programs can effectively target physical frailty and sarcopenia aiming to preserve mobility in vulnerable older people [7].

Rehabilitation for older adults should also consider psychosocial components like anxiety or loneliness [21]. Yamashita et al. noted that most people undergoing rehabilitation tend to set goals that are closely related to activity and participation. When the number of overlapping areas of frailty is limited, individual rehabilitation goals often center around community and social life. In cases of multifaceted individual frailty, the emphasis of rehabilitation goals primarily centers on targets related to mobility or self-care [22].

To address patient’s needs, the rehabilitation team should consist of a doctor with knowledge of GR, such as geriatricians and specialist rehabilitation experts, qualified nurses, physiotherapists, occupational therapists and social workers. A psychologist, pharmacist, dietician and speech therapist may also be involved, if required [8] (also see Fig. 1).

## 3 Future challenges

In contemporary times, rehabilitation offerings are heavily contingent on public funding, resulting in frequently standardized and economically oriented staffing approaches. This Mini-Review showcases the latest evidence on rehabilitation opportunities for older people with multimorbidity and frailty. There is growing evidence, that a person-centered and contextualized rehabilitation approach may offer benefits, not only in the context of preserving mobility, but especially targeting social participation. Modern rehabilitation aligns with the bio-psycho-social ICF model, emphasizing the individual definition of rehabilitation goals at the activity and participation level. The functional level should be considered secondary, with the evaluation of rehabilitation success based on the attainment of individual participation-oriented goals. The goal setting process need not be overly structured, but should be collaboratively determined with the patient through SDM aiming for a personalized approach. Further studies are warranted to evaluate objective outcome-measurement tools within the domains of activity and participation.

## Conflict of interest

Authors do not declare any financial conflict of interest
